# Variation in hospital caesarean section rates for women with at least one previous caesarean section: a population based cohort study

**DOI:** 10.1186/s12884-015-0609-x

**Published:** 2015-08-19

**Authors:** Kathrin Schemann, Jillian A. Patterson, Tanya A. Nippita, Jane B. Ford, Christine L. Roberts

**Affiliations:** Clinical and Population Perinatal Health Research, Kolling Institute of Medical Research, University of Sydney at Royal North Shore Hospital, St Leonards, NSW 2065 Australia; NSW Biostatistics Training Program, NSW Ministry of Health, North Sydney, NSW 2060 Australia; Department of Obstetrics and Gynaecology, Royal North Shore Hospital, Northern Sydney Local Health District, St Leonards, NSW 2065 Australia

## Abstract

**Background:**

Internationally, repeat caesarean sections make the largest contribution to overall caesarean section rates and inter-hospital variation has been reported. The aim of this study was to determine if casemix and hospital factors explain variation in hospital rates of repeat caesarean sections and whether these rates are associated with maternal and neonatal morbidity.

**Methods:**

This population-based record linkage study utilised data from New South Wales, Australia between 2007 and 2011. The study population included maternities with any previous caesarean section(s) and were singleton, cephalic and ≥37 weeks’ gestation (Robson Group 5). Multilevel regression models were used to examine variation in hospital rates of ‘planned repeat caesarean section’ and, among women who planned a vaginal birth, ‘intrapartum caesarean section’. We assessed associations between risk-adjusted hospital rates of planned and intrapartum caesarean sections and rates of casemix adjusted maternal and neonatal morbidity, postpartum haemorrhage and Apgar score <7 at five minutes.

**Results:**

Of 61894 maternities with a previous caesarean section in 81 hospitals, 82.1 % resulted in a caesarean section (72.7 % planned and 9.4 % unplanned intrapartum caesareans) and 17.9 % in vaginal birth. Observed hospital rates of planned caesarean sections ranged from 50.7 % to 98.4 %. Overall 49.0 % of between-hospital variation in planned repeat caesarean section rates was explained by patient (17.3 %) and hospital factors (31.7 %). Increased odds of planned caesarean section were associated with private hospital status and lower hospital propensity for vaginal birth after caesarean. There were no associations between hospital rates of planned repeat caesarean section and adjusted morbidity rates. Among women who intended a vaginal birth, the observed rates of intrapartum caesarean section ranged from 12.9 % to 71.9 %. In total, 27.5 % of between-hospital variation in rates of intrapartum caesarean section was explained by patient (19.5 %) and hospital factors (8.0 %). The adjusted morbidity rates differed among hospital intrapartum caesarean section rates, but were influenced by a few hospitals with outlying morbidity rates.

**Conclusions:**

Among women with at least one previous caesarean section, less than half of the variation in hospital caesarean section rates was explained by differences in hospital’s patient characteristics and practices. Strategies aimed at modifying caesarean section rates for these women should not affect morbidity rates.

## Background

Internationally, caesarean section rates have increased by 50 % or more over the last decade, with rates in the USA, UK and Australia peaking at 26.2 %, 31.3 % and 32.3 %, respectively [1–3]. The lack of availability of caesarean section in developing countries contributes to significant preventable maternal and perinatal morbidity and mortality [4]; yet in developed countries, rising caesarean section rates have not been accompanied by improved perinatal outcomes [5]. In 1985, the World Health Organization recommended a rate between 10 % and 15 % [6], and in 2009 acknowledged that it is important for the appropriate parturient to receive the optimal labour interventions, balancing the risks and benefits of each intervention [7].

Caesarean sections for women with at least one previous caesarean section make an important contribution to the historic rise in the overall caesarean section rate [8, 9]. In high income countries, Group 5 (multiparous women with at least one caesarean section and a single cephalic pregnancy at ≥37 weeks) [10] of the Robson classification for caesarean sections make the largest contribution to overall caesarean section rates [10–13]. This heterogenous group includes women with differing onsets of labour [10, 14–17], women with and without a previous vaginal delivery and women with one or more previous caesarean sections [14, 15, 18, 19]. To our knowledge, only one previous study examined adjusted hospital caesarean section rates for Robson Group 5 and identified large, unexplained variation between hospital rates for this group, despite adjustment for a limited number of case-mix factors (maternal age, country of birth of the mother, parity, maternal smoking, diabetes and hypertension) [11]. Variation in hospital caesarean section rates can be due to various factors including differences in patient characteristics or preferences, access to care, clinician behavior and hospital culture or policy. With sufficient data available on individual and hospital characteristics, variation due to known casemix and hospital factors can be accounted for, and the remaining ‘unexplained’ variation quantified. This ‘unexplained’ variation represents the contribution of unmeasured factors which result in women with similar characteristics having different outcomes depending on hospital of care [20, 21]. Unexplained variation in practice is important where it influences health care costs without improving outcomes, and raises questions about the appropriateness of particular hospital practices [22]. However, the previous study lacked information about differences in maternal and infant outcomes or evaluation of hospital characteristics that may contribute to the variation. Clinical factors such as offering trial of labour may also contribute to the variation in elective repeat caesarean section rates and subanalysis of this group by onset of labour has been suggested [17]. Therefore, the aims of this study were, among women with at least one previous caesarean section (Robson Group 5), a) to explore variation in hospital rates of planned and intrapartum repeat caesarean section by intended mode of birth; b) to determine whether case-mix and hospital factors explain the variation between hospital rates and c) to examine the association between hospital rates of planned and intrapartum caesarean sections with maternal and neonatal morbidity outcomes.

## Methods

### Study population

The study population included multiparous women with at least one previous caesarean section and a single, cephalic pregnancy at ≥37 weeks’ gestation (Robson Group 5, [10]), who gave birth in New South Wales (NSW) in 2007 – 2011. NSW is Australia’s most populated state with 7 million residents and 95 000 births per annum (32 % of all Australian births) [23]. This study was restricted to births occurring in hospitals having at least 50 births per annum and performing at least 10 caesarean sections per annum. The analyses of intrapartum caesarean section rates following a trial of labour were additionally restricted to hospitals with at least 20 women undergoing a trial of labour during the study period to ensure that only hospitals that offered vaginal birth after caesarean section were included, and to provide sufficient data for modeling of the hospital rate of intrapartum caesarean section.

### Data sources and sampling

Data were obtained from two NSW population databases, the Perinatal Data Collection (PDC) and the Admitted Patient Data Collection (APDC). The PDC is a legislated population-based surveillance system covering all live births and stillbirths of at least 20 weeks gestation or 400 grams birth weight in NSW (subsequently referred to as ‘births’). Information in the PDC is collected by the attending doctor or midwife and includes maternal characteristics, medical and obstetric information as well as infant outcomes. The APDC represents a census of all NSW public and private hospital discharges and includes patient characteristics and hospitalisation-related information. Up to 20 diagnoses and procedures in each hospital record are coded according to the 10^th^ revision of the International Classification of Disease, Australian Modification (ICD-10-AM) and the Australian Classification of Health Interventions [24]. Trained clinical coders obtain diagnosis and procedure information by reviewing the entire medical record. The NSW Centre for Health Record Linkage carried out probabilistic record linkage between the two databases, with linkage proportions over 98 % [25], prior to providing the researchers with de-identified records for analysis.

### Outcome variables

The primary outcomes were hospital rates of ‘planned caesarean sections’ and, among women who planned a vaginal birth ‘intrapartum caesarean sections’. ‘Planned caesarean sections’ included women who had at least one previous caesarean section and subsequently had a caesarean section, with no labour or went into spontaneous labour before their planned date [26]. Women who planned a vaginal birth included all other women with spontaneous, augmented or induced labour, regardless of eventual mode of delivery.

### Explanatory variables

The available explanatory variables were categorised into case-mix factors (Table 1) and hospital factors. Maternal age was treated as a continuous variable in analyses. Two area-based variables, socio-economic status using the index of education and occupation based on 2011 census data [27] by postcode of residence and the rate of overweight/obesity based on local health district and year [28] were assigned to each individual record and used as explanatory variables. Hospital factors included birth volume, hospital status (private; public with primary obstetric training; public with secondary obstetric training (large district and rural hospitals that host obstetric registrars); and other (non-training public hospitals)), level of perinatal care (NICU, CPAP, other) and hospital location (urban/rural) [29]. Hospital rates of obstetric transfusions; instrumental birth (forceps and vacuum assisted); caesarean sections performed under general anaesthetic and births where regional analgesia was used (as indicators of anaesthetic services) were also considered. The hospital rate of all hospital births that were low risk births (rate of deliveries of singleton, cephalic pregnancies at term without hypertension, placental conditions, diabetes or other chronic disease and no previous perinatal death, referred to as low risk rate) and hospital usage of oxytocin for induction and/or augmentation among women with previous caesarean section were also used. Additionally, the hospital rate of vaginal delivery for the birth following a primary caesarean section for breech presentation was used as a proxy for hospital predisposition to carry out a vaginal birth after caesarean (referred to as ‘hospital propensity towards VBAC’). Variables from the birth record are considered to be reliably reported [30–33] and the coding of hospital diagnoses and procedures has previously been validated [34–37].

**Table 1:** Case-mix characteristics of the study population, NSW, 2007–2011. The study population consists of multiparous women with a singleton cephalic-presenting infant at ≥37 weeks gestation with at least one previous caesarean section

Variables and levels		All deliveries	Any repeat caesarean^a^	Planned repeat CS	Intrapartum repeat CS	Vaginal birth after caesarean	Planned vaginal birth^b^
		N=61894 (column %)	*N* = 50819 (column %)	N=45006 (column %)	N=5813 (column %)	*N* = 11075 (column %)	N=16888 (column %)
Maternal age	Under 20	314 (0.5)	248 (0.5)	211 (0.5)	37 (0.6)	66 (0.6)	103 (0.6)
	20 to 34	36626 (59.2)	29472 (58.0)	25843 (57.4)	3629 (62.4)	7154 (64.6)	10783 (63.9)
	35+	24954 (40.3)	21099 (41.5)	18952 (42.1)	2147 (36.9)	3855 (34.8)	6002 (35.5)
Country of birth	Australia or New Zealand	45012 (72.9)	37018 (73.0)	32947 (73.4)	4071 (70.2)	7994 (72.3)	12065 (71.6)
	Europe or North America	3554 (5.8)	2921 (5.8)	2575 (5.7)	346 (6.0)	633 (5.7)	979 (5.8)
	Other	13177 (21.3)	10750 (21.2)	9368 (20.9)	1382 (23.8)	2427 (22.0)	3809 (22.6)
Model of care	Public patient in public hospital	36059 (58.2)	28020 (55.1)	24079 (53.5)	3941 (67.8)	8039 (72.6)	11980 (70.9)
	Private patient in public hospital	5544 (9.0)	4667 (9.2)	4201 (9.3)	466 (8.0)	877 (7.9)	1343 (8.0)
	Private patient in private hospital	20291 (32.8)	18132 (35.7)	16726 (37.2)	1406 (24.2)	2159 (19.5)	3565 (21.1)
Smoking in pregnancy		6174 (10.0)	4599 (9.1)	3947 (8.8)	652 (11.2)	1575 (14.2)	2227 (13.2)
Diabetes (pre-existing or gestational)		5233 (8.5)	4598 (9.0)	4166 (9.3)	432 (7.4)	635 (5.7)	1067 (6.3)
Hypertensive disorders		3327 (5.4)	2842 (5.6)	2510 (5.6)	332 (5.7)	485 (4.4)	817 (4.8)
Other chronic medical conditions^c^		788 (1.3)	709 (1.4)	625 (1.4)	84 (1.4)	79 (0.7)	163 (1.0)
Parity 2 or more (compared to parity 1)		23911 (38.7)	18516 (36.5)	17132 (38.2)	1384 (23.8)	5395 (48.8)	6779 (40.2)
2 or more previous caesarean sections		13554 (22.0)	13353 (26.4)	12831 (28.6)	522 (9.0)	201 (1.9)	723 (4.3)
Labour/vaginal birth prior to first caesarean		5819 (9.4)	3851 (7.6)	3383 (7.5)	468 (8.1)	1968 (17.8)	2436 (14.4)
Previous vaginal birth after caesarean		4130 (6.7)	1121 (2.2)	834 (1.9)	287 (4.9)	3009 (27.2)	3296 (19.5)
Previous 3^rd^ or 4^th^ degree perineal tear		551 (0.9)	402 (0.8)	372 (0.8)	30 (0.5)	149 (1.3)	179 (1.1)
Previous perinatal death		1000 (1.6)	825 (1.6)	752 (1.7)	73 (1.3)	175 (1.6)	248 (1.5)
Non-CS uterine scar		421 (0.7)	403 (0.8)	377 (0.8)	26 (0.4)	18 (0.2)	44 (0.3)
Assisted reproductive technology use, last 12 months		1914 (3.1)	1594 (3.1)	1447 (3.2)	147 (2.5)	320 (2.9)	467 (2.8)
Inter-pregnancy interval (months)	0-17	21126 (34.1)	17082 (33.6)	15136 (33.6)	1946 (33.5)	4044 (36.5)	5990 (35.5)
	18-59	27761 (44.9)	23069 (45.4)	20617 (45.8)	2452 (42.2)	4692 (42.4)	7144 (42.3)
	60+	4903 (7.9)	4066 (8.0)	3605 (8.0)	461 (7.9)	837 (7.6)	1298 (7.7)
	Unknown	8104 (13.1)	6602 (13.0)	5648 (12.5)	954 (16.4)	1502 (13.6)	2456 (14.5)
Placental conditions^d^		1079 (1.7)	990 (1.9)	807 (1.8)	183 (3.1)	89 (0.8)	272 (1.6)
Gestational age (weeks)	37	4597 (7.4)	3870 (7.6)	3320 (7.4)	550 (9.5)	727 (6.6)	1277 (7.6)
	38	19452 (31.4)	17579 (34.6)	16389 (36.4)	1190 (20.5)	1873 (16.9)	3063 (18.1)
	39	25143 (40.6)	21915 (43.1)	20366 (45.3)	1549 (26.6)	3228 (29.1)	4777 (28.3)
	40	9016 (14.6)	5367 (10.6)	3782 (8.4)	1585 (27.3)	3649 (32.9)	5234 (31.0)
	41+	3686 (6.0)	2088 (4.1)	1149 (2.6)	939 (16.2)	1598 (14.4)	2537 (15.0)
Birth weight for gestational age	Small (<10^th^ percentile)	5,501 (8.9)	4110 (8.1)	3501 (7.8)	609 (10.5)	1391 (12.6)	2000 (11.8)
	Appropriate	49453 (79.9)	40540 (79.8)	35962 (79.9)	4578 (78.8)	8913 (80.5)	13491 (79.9)
	Large (>90^th^ percentile)	6927 (11.2)	6158 (12.1)	5533 (12.3)	625 (10.8)	769 (6.9)	1394 (8.3)
Stillbirth		60 (0.1)	30 (0.1)	23 (0.1)	7 (0.1)	30 (0.3)	37 (0.2)
Severe maternal morbidity		654 (1.9)	526 (1.9)	395 (1.6)	131 (4.0)	128 (2.1)	259 (2.8)
Severe neonatal morbidity		1275 (2.1)	1058 (2.1)	851 (1.9)	207 (3.6)	217 (2.0)	424 (2.5)
Postpartum haemorrhage		2847 (4.6)	1666 (3.3)	1301 (2.9)	365 (6.3)	1181 (10.7)	1546 (9.2)
Apgar score at five minutes <7		520 (0.8)	371 (0.7)	261 (0.6)	110 (1.9)	149 (1.4)	259 (1.5)

CS = caesarean section

^a^Includes planned repeat CS and intrapartum repeat CS

^b^Includes intrapartum repeat caesarean section and vaginal birth after caesarean section

^c^Included renal, cardiac, asthma/COPD, autoimmune, thyroid and inflammatory bowel disease

^d^Included morbidly adherent placenta, placental abruption, placenta praevia, antepartum haemorrhage

### Statistical analyses

Descriptive statistics for continuous variables and frequency tables for categorical variables were used to assess the distribution of the explanatory variables. Mean and standard deviation are reported for normally distributed continuous variables whereas median and inter-quartile ranges are reported for non-normally distributed continuous variables. Observed hospital rates were reported for a) ‘planned caesarean sections’ (among all women with at least one previous caesarean, Robson Group 5) and b) ‘intrapartum caesarean sections’ (among all women with at least one previous caesarean who had a trial of labour for a planned vaginal birth).

For each of the two outcomes above, observed hospital rates were compared. Then, multivariable, multilevel binomial logistic regression models were constructed with a manual backward stepwise approach, with a random intercept for hospital to account for clustering of observations from the same hospital and with a shrinkage factor to allow for inclusion of hospitals with small sample size. Models were fitted, progressively adjusting for case-mix and hospital factors, as described previously [38]. Briefly, the first model included only the random hospital effect (hospital intercepts), and produced ‘unadjusted rates’ accounting only for clustering within hospitals, with rates for smaller hospitals ‘shrunken’ towards the average rate. Thereafter models were sequentially adjusted for case-mix factors (model 2) and case-mix and hospital factors (model 3).To illustrate the differences in hospital repeat caesarean section rates after each step of adjustment, the risk-adjusted hospital rates with 95 % confidence intervals were plotted. The relative contribution of each step of adjustment to the overall reduction in variation in hospital caesarean section rates was quantified by calculating the difference between the hospital variation of the current and preceding models as a proportion of the unadjusted model’s hospital variation.

### Assessment of associations of planned and intrapartum hospital caesarean section rates with maternal or neonatal morbidity

The associations between hospital planned and intrapartum caesarean rates and hospital morbidity rates were assessed. Morbidities included severe maternal and neonatal morbidity, postpartum haemorrhage and infant Apgar score below 7 at five minutes. Severe maternal and neonatal morbidity were determined using validated composite outcome indicators that include both life-threatening conditions (e.g. respiratory failure, cerebrovascular haemorrhage, shock and cardiac arrest) and procedures associated with severe morbidity (e.g. mechanical ventilation, blood transfusion, acute dialysis and surgical procedures) [35, 39]. Postpartum haemorrhage and Apgar score are accurately reported in these data [30, 40]. A multilevel logistic regression approach was used. Hospitals were divided into quintiles according to their risk-adjusted caesarean section rates. The quintiles were then used as an additional categorical variable to predict case-mix adjusted morbidity rates. Case-mix adjusted hospital rates of maternal and neonatal morbidity within each caesarean section rate quintile were then averaged and the estimated adjusted odds ratios of morbidity for each quintile were compared. The risk-adjusted hospital caesarean section rates were also plotted against the case-mix adjusted rates of maternal and neonatal morbidity.

All statistical analyses were conducted using SAS Enterprise Guide statistical software (release 5.1 © 2012, SAS Institute Inc., Cary, NC, USA).

This study was approved by the NSW Population and Health Services Research Ethics Committee.

## Results

From 2007 to 2011 there were 63316 singleton cephalic, term births in NSW among mothers with at least one previous caesarean section (Robson Group 5). Among all maternities, the proportions of births in Robson Group 5 increased from 12.7 % in 2007 to 14.1 % in 2011. After exclusions (739 from ineligible hospitals and 683 with missing data), 61894 maternities from 81 hospitals were included in the analysis. Overall, there were 51388 (82.1 %) women who had a further caesarean section; including 45006 (72.7 %) deliveries with a planned caesarean section. Of the 16888 women intending to have a vaginal birth, 34.4 % had an intrapartum caesarean section and 65.6 % had a vaginal birth.

The distributions of key characteristics among women with at least one previous caesarean section are presented in Table 1. Women with a planned caesarean section tended to be older, private patients, have more medical and obstetric conditions and two or more previous caesarean sections than women with a trial of labour. Among women with a trial of labour for a planned vaginal birth, those who had intrapartum caesarean sections were more likely to be private patients, have two or more previous caesarean sections and have not previously experienced labour or vaginal birth before their first caesarean or a successful vaginal birth after caesarean compared to women with vaginal birth (Table 1).

Of the 81 hospitals, 16 (19.8 %) were private as opposed to public, 47 (58.0 %) were regional as opposed to metropolitan and 31 (38.3 %) provided primary or secondary obstetric training as opposed to not providing obstetric training. Over the study period, the median annual hospital volume of singleton cephalic deliveries at term by multiparous women with a previous caesarean scar was 119 (Inter quartile range: 31–223). The mean hospital instrumental birth rate for all maternities with labour was 14.6 % (standard deviation: 5.6 %). The mean hospital rate of propensity towards VBAC was 63.2 % (standard deviation: 19.2 %).

### Variation in hospital planned caesarean section rates

**Fig. 1:**
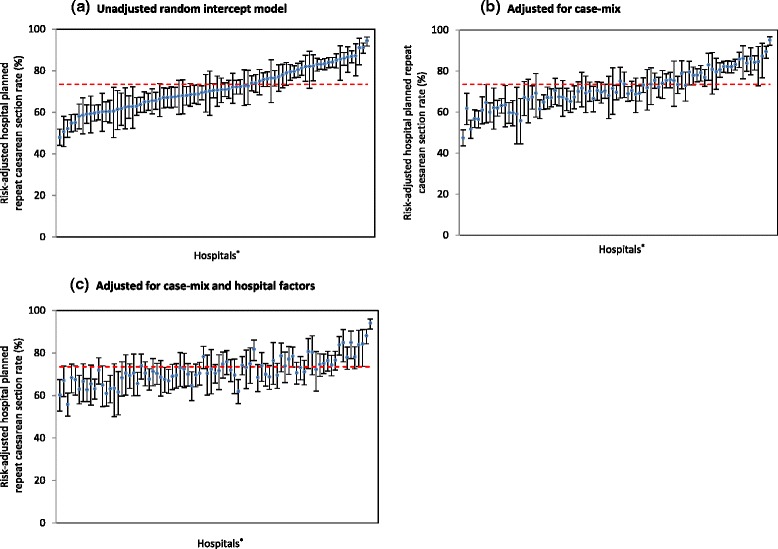
Hospital planned repeat caesarean section rates in NSW, 2007–2011. ^*^ The data points represent the rates of planned repeat caesarean section among multiparous women with a singleton, cephalic-presenting infant of ≥37 weeks gestation in 81 hospitals, ordered from lowest to highest unadjusted hospital CS rate. The vertical error bars indicate the 95 % confidence intervals for each estimate. Dashed horizontal lines indicate the mean risk-adjusted planned caesarean section rate

Among women with at least one previous caesarean, the observed hospital planned caesarean section rates for the 81 hospitals ranged from 50.7 % to 98.4 %. The unadjusted model had hospital rates ranging from 47.9 % to 94.4 % and 52 of 81 hospitals had rates that differed from the state average (unadjusted model; Fig. 1a). After adjusting for case-mix factors, the unexplained variation between hospitals was reduced by 17.3 % with the adjusted hospital planned caesarean section rates ranging from 47.5 % to 95.0 % (Fig. 1b). However, 42 of 81 hospitals differed from the state average. Maternal medical and prior pregnancy complications were positively associated with planned caesarean section (Table 2). Further adjustment using hospital-level factors explained an additional 31.7 % of the variation between hospital planned caesarean section rates, with adjusted rates ranging from 55.8 % to 94.1 % (Fig. 1c; Table 2). Birthing in hospitals that were private, had low propensity towards VBAC and had a high rate of low risk maternities was associated with higher odds of planned caesarean section (Table 2). There was a tendency for hospitals that used oxytocin to induce and/or augment women with a previous caesarean section to have lower odds of planned caesarean section (OR: 0.95, 95 % CI: 0.90, 1.01; Table 2). Overall the final model adjusting for casemix and hospital factors explained 49.0 % of the variation between hospital planned repeat caesarean section rates, mostly due to hospital factors, but 23 of 81 hospitals had adjusted rates that differed from the state average.

**Table 2:** Predictive factors for planned repeat caesarean section, NSW, 2007–2011. Two models were fitted progressively adjusting for case-mix and hospital factors

Variable and categories	Case-mix model	Hospital model
	OR (95 % CI)	OR (95 % CI)
Maternal age (years)	1.02 (1.02, 1.03)	1.02 (1.02, 1.03)
Country of birth		
Australia or New Zealand	1.00	1.00
Europe or North America	0.83 (0.76, 0.91)	0.83 (0.76, 0.91)
Other	0.87 (0.82, 0.93)	0.88 (0.83, 0.93)
Smoking	0.91 (0.85, 0.98)	0.92 (0.85, 0.99)
Socio-economic status^a^		
1st quintile (high)	1.27 (1.14, 1.42)	1.28 (1.15, 1.43)
2nd quintile	1.17 (1.06, 1.30)	1.18 (1.07, 1.31)
3rd quintile	1.18 (1.07, 1.30)	1.18 (1.07, 1.30)
4th quintile	1.01 (0.93, 1.10)	1.01 (0.93, 1.10)
5th quintile (low)	1.00	1.00
Diabetes (pre-existing or gestational)	1.27 (1.17, 1.38)	1.28 (1.18, 1.39)
Hypertensive disorders	1.10 (1.00, 1.21)	1.11 (1.01, 1.22)
Other chronic medical conditions^b^	1.42 (1.16, 1.74)	1.43 (1.17, 1.74)
Placental conditions^c^	1.11 (0.94, 1.30)	1.11 (0.94, 1.30)
Parity 2 or more (versus parity 1)	2.09 (1.97, 2.22)	2.10 (1.98, 2.22)
Labour/vaginal birth prior to index caesarean	0.28 (0.26, 0.31)	0.28 (0.26, 0.31)
Previous vaginal birth after caesarean	0.05 (0.04, 0.05)	0.05 (0.04, 0.05)
Previous 3^rd^ or 4^th^ degree perineal tear	2.44 (1.93, 3.07)	2.44 (1.93, 3.07)
Previous perinatal death	2.66 (2.20, 3.21)	2.66 (2.20, 3.20)
Non-CS uterine scar	2.20 (1.55, 3.12)	2.21 (1.56, 3.13)
Assisted reproductive technology use (last 12 months)	0.81 (0.72, 0.91)	0.81 (0.71, 0.91)
Inter-pregnancy interval (months)		
0-17	0.90 (0.86, 0.95)	0.90 (0.86, 0.95)
18-59	1.00	1.00
60+	1.05 (0.96, 1.14)	1.05 (0.96, 1.14)
Unknown	0.69 (0.64, 0.73)	0.69 (0.64, 0.73)
Estimated gestational age (weeks)		
37	0.57 (0.52, 0.62)	0.57 (0.52, 0.62)
38	1.14 (1.08, 1.21)	1.14 (1.08, 1.21)
39	1.00	1.00
40	0.18 (0.17, 0.20)	0.18 (0.17, 0.20)
41+	0.14 (0.12, 0.15)	0.14 (0.12, 0.15)
Small for gestational age (<10^th^ percentile)	0.77 (0.71, 0.84)	0.77 (0.71, 0.84)
Large for gestational age (>90^th^ percentile)	1.47 (1.38, 1.57)	1.47 (1.38, 1.57)
Stillbirth	0.24 (0.13, 0.46)	0.25 (0.13, 0.46)
Repeat caesarean rate after nulliparous breech caesarean (Propensity towards vaginal birth after caesarean section)	-	0.95 (0.93, 0.96)
Oxytocin induction and/or augmentation for women with previous caesarean	-	0.95 (0.90, 1.01)
Low risk rate among all birth	-	1.18 (1.05, 1.32)
Level of hospital care	-	
Public with primary training		1.00
Public with secondary training	-	1.30 (0.91, 1.85)
Public, other	-	1.28 (0.89, 1.82)
Private	-	1.80 (1.24, 2.61)

^a^Index of Education and Occupation published by the Australian Bureau of Statistics based on postcode [27]

^b^Included renal, cardiac, asthma/c obstructive pulmonary, autoimmune, thyroid and inflammatory bowel disease

^c^Included morbidly adherent placenta, placental abruption, placenta praevia, antepartum haemorrhage

### Variation in hospital intrapartum caesarean section rates following a trial of labour

**Fig. 2:**
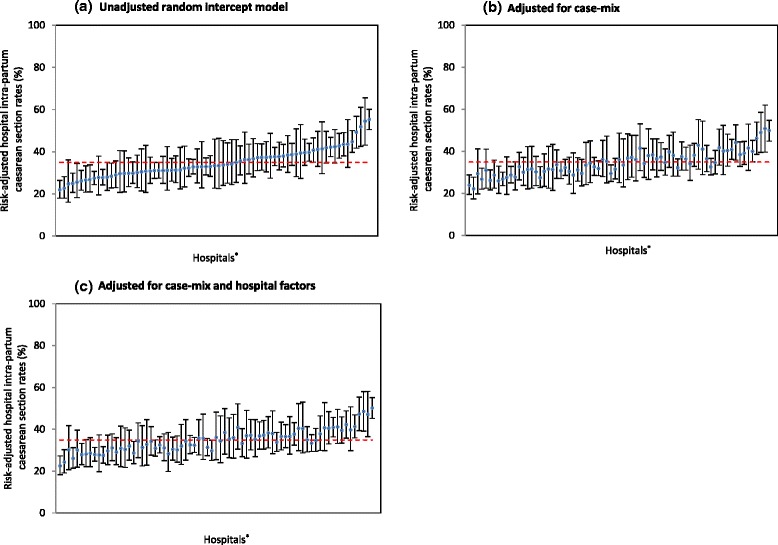
Hospital intrapartum repeat caesarean section rates in NSW, 2007–2011. ^*^ The data points represent the rates of intrapartum repeat caesarean section among multiparous women with a singleton, cephalic-presenting infant of ≥37 weeks gestation undergoing a trial of labour in 73 hospitals, ordered from lowest to highest unadjusted hospital CS rate. The vertical error bars indicate the 95 % confidence intervals for each estimate. Dashed horizontal lines indicate the mean risk-adjusted planned caesarean section rates

Among women with at least one previous caesarean who had a trial of labour for a planned vaginal birth, the observed hospital rates of intrapartum caesarean section ranged from 12.9 % to 71.9 %. Unadjusted hospital rates of intrapartum caesarean section following a trial of labour ranged from 21.9 % to 55.3 % and 20 of 73 hospitals had rates that differed from the state average (Fig. 2a). After adjusting for case-mix factors, the unexplained variation between hospitals was reduced by 19.5 % with the adjusted hospital intrapartum caesarean section rates ranging from 22.2 % to 50.9 % and 16 of 73 hospitals being different from the state average (Fig. 2b). Additional adjustment for hospital factors further reduced the unexplained variation by 8.0 % with adjusted rates ranging from 22.5 % to 50.2 %, Fig. 2c). The details of these models are presented in Table 3. Hospitals with generally higher instrumental delivery rates had lower odds of having intrapartum caesarean section (OR: 0.82, 95 % CI: 0.71, 0.93; Table 3). Overall the final model explained 27.5 % of the variation between hospital intrapartum caesarean section rates but 14 of 73 hospitals differed from the state average.

**Table 3:** Predictive factors for intrapartum caesarean section among women with a trial of labour for a planned vaginal birth, NSW, 2007–2011. Two models were fitted progressively adjusting for case-mix and hospital factors

Variable and categories	Case-mix model	Hospital model
	OR (95 % CI)	OR (95 % CI)
Maternal age (years)	1.02 (1.01, 1.03)	1.02 (1.02, 1.03)
Country of birth		
Australia or New Zealand	1.00	1.00
Europe or North America	0.86 (0.74, 1.00)	0.86 (0.74, 1.00)
Other	1.17 (1.06, 1.29)	1.17 (1.06, 1.29)
Smoking	1.15 (1.03, 1.29)	1.14 (1.02, 1.29)
Diabetes (pre-existing or gestational)	1.27 (1.10, 1.46)	1.27 (1.10, 1.46)
Hypertensive disorders	1.31 (1.12, 1.53)	1.31 (1.12, 1.53)
Other chronic medical conditions^a^	1.95 (1.38, 2.73)	1.93 (1.37, 2.71)
Parity 2 or more (versus parity 1)	0.70 (0.63, 0.78)	0.70 (0.63, 0.78)
Labour/vaginal birth prior to index caesarean	0.48 (0.42, 0.56)	0.48 (0.42, 0.55)
Previous vaginal birth after caesarean	0.16 (0.14, 0.19)	0.16 (0.14, 0.19)
Previous perinatal death	1.80 (1.32, 2.46)	1.79 (1.31, 2.44)
Non-CS uterine scar	2.78 (1.41, 5.48)	2.75 (1.39, 5.44)
Assisted reproductive technology use (last 12 months)	0.55 (0.44, 0.68)	0.55 (0.45, 0.68)
Inter-pregnancy interval (months)		
0-17	0.92 (0.85, 1.00)	0.92 (0.85, 1.00)
18-59	1.00	1.00
60+	1.22 (1.06, 1.40)	1.21 (1.06, 1.40)
Unknown	0.83 (0.74, 0.92)	0.82 (0.74, 0.92)
Placental conditions^b^	4.26 (3.22, 5.65)	4.26 (3.22, 5.65)
Estimated gestational age (weeks)		
37	1.58 (1.37, 1.82)	1.58 (1.38, 1.82)
38	1.30 (1.18, 1.44)	1.30 (1.18, 1.45)
39	1.00	1.00
40	0.94 (0.86, 1.03)	0.94 (0.86, 1.03)
41+	1.41 (1.26, 1.58)	1.41 (1.26, 1.57)
Large for gestational age (>90^th^ percentile)	1.71 (1.53, 1.90)	1.71 (1.53, 1.91)
Stillbirth	0.26 (0.11, 0.62)	0.25 (0.10, 0.62)
Repeat caesarean rate after nulliparous breech caesarean (Propensity towards vaginal birth after caesarean section)	-	1.04 (1.02, 1.07)
Instrumental delivery rate	-	0.82 (0.71, 0.93)

^a^Included renal, cardiac, asthma/COPD, autoimmune, thyroid and inflammatory bowel disease

^b^Included morbidly adherent placenta, placental abruption, placenta praevia, antepartum haemorrhage

### Associations with maternal and neonatal morbidities

**Fig. 3:**
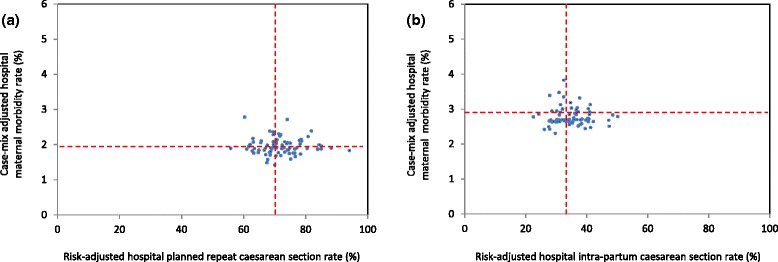
Severe maternal morbidity and risk-adjusted hospital (**a**) planned and (**b**) intrapartum caesarean section rates. Dashed lines indicate the respective mean risk-adjusted caesarean and case-mix adjusted morbidity rates. The study population consisted of multiparous women with a previous caesarean section and a singleton, cephalic-presenting infant of ≥37 weeks gestation in NSW, 2007–2011

**Fig. 4:**
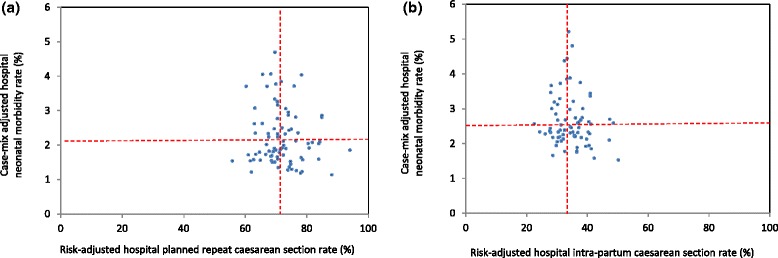
Severe neonatal morbidity and risk-adjusted hospital (**a**) planned and (**b**) intrapartum caesarean section rates. Dashed lines indicate the respective mean risk-adjusted caesarean and case-mix adjusted morbidity rates. The study population consisted of multiparous women with a previous caesarean section and a singleton, cephalic-presenting infant of ≥37 weeks gestation in NSW, 2007–2011

**Fig. 5:**
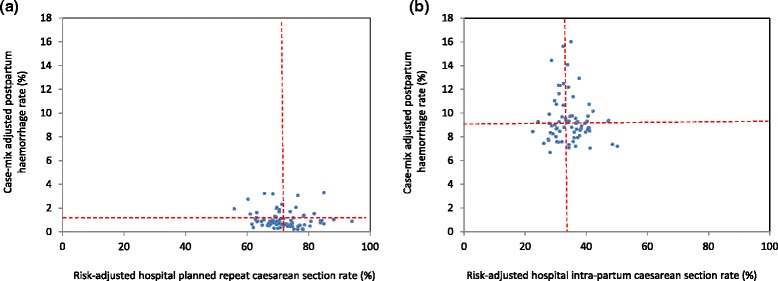
Postpartum haemorrhage and risk-adjusted hospital (**a**) planned and (**b**) intrapartum caesarean section rates. Dashed lines indicate the respective mean risk-adjusted caesarean and case-mix adjusted morbidity rates. The study population consisted of multiparous women with a previous caesarean section and a singleton, cephalic-presenting infant of ≥37 weeks gestation in NSW, 2007–2011

**Fig. 6:**
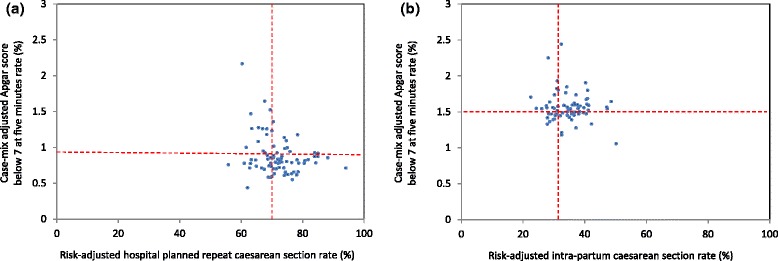
Apgar score below 7 at five minutes and risk-adjusted hospital (**a**) planned and (**b**) intrapartum caesarean section rates. Dashed lines indicate the respective mean risk-adjusted caesarean and case-mix adjusted morbidity rate. The study population consisted of multiparous women with a previous caesarean section and a singleton, cephalic-presenting infant of ≥37 weeks gestation in NSW, 2007–2011

Overall, when compared to planned caesarean sections, morbidity rates (severe maternal and neonatal morbidity, postpartum haemorrhage and Apgar score at five minutes less than 7) were higher for women undergoing a trial of labour for a planned vaginal birth (Table 1). Highest rates of adverse outcomes were primarily among women with an intrapartum caesarean section, with the notable exception of postpartum haemorrhage which was highest among women with a vaginal birth. However, in Australia the threshold for postpartum haemorrhage is lower (500 ml) for vaginal birth than for caesarean section (750 ml)[24]. For planned repeat caesarean sections, case-mix adjusted hospital rates of severe maternal morbidity, ranged from 1.4 % to 2.8 % (Fig. 3a) and case-mix adjusted hospital rates of severe neonatal morbidity ranged from 1.1 % to 4.7 % (Fig. 4a). Adjusted hospital rates of postpartum haemorrhage ranged from 0.2 % to 3.3 % for all births (Fig. 5a) and adjusted rates of Apgar score below 7 at five minutes ranged from 0.4 % to 2.2 % (Fig. 6a). There were no associations between hospital planned caesarean section rate quintiles and any of the morbidity measures and no specific patterns indicated in the scatter plots (Figs. 3a, 4a, 5a, 6a; Table 4).

**Table 4:** Case-mix adjusted morbidity rates across quintiles of hospital planned caesarean section rates in NSW, 2007–2011

Hospital quintiles	Maternal morbidity		Neonatal morbidity		Postpartum haemorrhage		Apgar score at 5 minutes <7	
	Rate (95 % CI)	OR (95 % CI)	Rate (95 % CI)	OR (95 % CI)	Rate (95 % CI)	OR (95 % CI)	Rate (95 % CI)	OR (95 % CI)
Planned repeat caesarean								
1st quintile (low)	2.1 (1.8, 2.4)	0.96 (0.70, 1.32)	2.4 (1.4, 3.4)	0.86 (0.58, 1.29)	1.6 (0.7, 2.5)	1.16 (0.69, 1.96)	1.1 (0.6, 1.7)	0.96 (0.70, 1.32)
2nd quintile	1.9 (1.5, 2.2)	0.78 (0.57, 1.08)	2.2 (1.4, 3.1)	0.97 (0.66, 1.43)	1.2 (0.2, 2.1)	0.97 (0.58, 1.62)	0.9 (0.6, 1.2)	0.78 (0.57, 1.08)
3rd quintile	2.1 (1.8, 2.3)	1.00	2.4 (1.7, 3.1)	1.00	1.1 (0.6, 1.6)	1.00	0.9 (0.7, 1.1)	1.00
4th quintile	1.9 (1.5, 2.3)	0.84 (0.61, 1.14)	2.2 (1.3, 3.1)	0.86 (0.58, 1.28)	0.9 (0.2, 1.5)	0.71 (0.42, 1.20)	0.7 (0.6, 0.9)	0.84 (0.61, 1.14)
5th quintile (high)	1.9 (1.7, 2.1)	1.02 (0.73, 1.43)	2.3 (1.2, 3.5)	0.73 (0.48, 1.13)	1.0 (0.5, 1.6)	0.83 (0.48, 1.43)	0.9 (0.7, 1.1)	1.02 (0.73, 1.43)
ALL	2.0 (1.8, 2.2)	–	2.3 (1.4, 3.2)	–	1.1 (0.4, 1.9)	–	0.9 (0.5, 1.3)	–

For births following a trial of labour, adjusted hospital rates of severe maternal and neonatal morbidity ranged from 2.3 % to 3.8 % and 1.5 % to 5.2 %, respectively (Figs. 3b and 4b). Hospital rates of postpartum haemorrhage, adjusted for case-mix factors, ranged from 6.7 % to 16.0 % for all deliveries following a trial of labour (Fig. 5b). The adjusted maternal morbidity rate was higher for quintile 2 of the hospital intrapartum caesarean section rates when compared to quintile 3, whereas adjusted neonatal morbidity and postpartum haemorrhage rates were lower for quintiles 1, 4 and 5 compared to quintile 3 of hospital intrapartum caesarean section rates (Table 5). A similar trend was observed for adjusted rates of Apgar score below 7 at five minutes (Fig. 6b). A few hospitals with outlying case-mix adjusted morbidity rates caused the differences in morbidity rates among quintiles of hospital intrapartum caesarean section rates (Figs. 4b and 5b).

**Table 5:** Case-mix adjusted morbidity rates across quintiles of hospital intrapartum caesarean section rates in NSW, 2007–2011

Hospital quintiles	Maternal morbidity		Neonatal morbidity		Postpartum haemorrhage		Apgar score at 5 minutes <7	
	Rate (95 % CI)	OR (95 % CI)	Rate (95 % CI)	OR (95 % CI)	Rate (95 % CI)	OR (95 % CI)	Rate (95 % CI)	OR (95 % CI)
Intrapartum caesarean								
1st quintile (low)	2.8 (2.4, 3.2)	0.95 (0.61, 1.49)	2.7 (2.0, 3.3)	0.65 (0.43, 0.99)	9.0 (7.6, 10.4)	0.73 (0.55, 0.97)	1.5 (1.3, 1.7)	0.96 (0.70, 1.32)
2nd quintile	3.3 (2.9, 3.6)	1.56 (1.03, 2.35)	3.0 (2.2, 3.9)	0.70 (0.46, 1.08)	12.0 (9.6, 14.3)	0.94 (0.72, 1.23)	1.8 (1.3, 2.2)	0.78 (0.57, 1.08)
3rd quintile	2.7 (2.6, 2.9)	1.00	3.6 (2.3, 4.9)	1.00	11.4 (9.0, 13.8)	1.00	1.5 (1.3, 1.7)	1.00
4th quintile	2.8 (2.5, 3.1)	0.15 (0.70, 1.88)	2.5 (1.8, 3.1)	0.55 (0.34, 0.88)	9.4 (7.7, 11.0)	0.72 (0.54, 0.98)	1.5 (1.4, 1.6)	0.84 (0.61, 1.14)
5th quintile (high)	2.7 (2.6, 2.9)	1.04 (0.65, 1.67)	2.3 (1.5, 3.0)	0.47 (0.29, 0.77)	8.8 (7.3, 10.3)	0.66 (0.46, 0.92)	1.5 (1.2, 1.8)	1.02 (0.73, 1.43)
ALL	2.9 (2.5, 3.3)	–	2.8 (1.9, 3.8)	–	10.3 (7.9, 12.6)	–	1.6 (1.3, 1.9)	–

## Discussion

Overall in NSW between 2007 and 2011, 82.1 % of women with at least one previous caesarean section and a singleton, cephalic infant at term had a further caesarean section, and for the majority of women it was a planned caesarean section (72.7 %). A trial of labour was relatively uncommon (27.3 %), although 65.6 % of those women attempting a trial of labour had a successful vaginal birth after caesarean. This finding suggests that even though not many women are selected for a trial of labour, the ones selected are good candidates for vaginal birth after caesarean. Consistent with other studies, both analyses in this study found that the strongest predictive factors for a vaginal birth after caesarean were having a previous vaginal birth after caesarean and having experienced labour/vaginal birth before the index caesarean [41]. Case-mix and hospital factors explained about half (49 %) of the variation in hospital rates of planned repeat caesarean sections and just over a quarter (27.5 %) of between hospital variation in intrapartum caesarean section rates. After final adjustment, 23 of 81 (28.4 %) and 14 of 73 (19.2 %) of hospitals had rates that differed from the state average for planned and intrapartum repeat caesarean sections, respectively. There were no associations between quintiles of hospital planned caesarean section rates and morbidity outcomes, but four outlying observations resulted in some associations between quintiles of hospital intrapartum caesarean section rates and morbidity outcomes. A triangular pattern was evident, with the second and third quintiles of hospital intrapartum caesarean section rates having the highest morbidity rates.

To our knowledge this is the first study to examine the variation in caesarean section rates among multiparous women with at least one previous caesarean section with a singleton cephalic fetus at term by intended mode of birth and to explore their maternal and neonatal outcomes. This study utilised large, linked population health datasets with reliably identified and validated variables used for risk adjustment. Multi-level modelling accounted for similarities of births within hospitals and inclusion of a shrinkage factor allowed inclusion of hospitals with small sample size. However, limitations include the lack of information on individual patient attitudes and physician decision-making processes. Different hospital reporting practices may also contribute to the observed hospital variation and this could not be further investigated in this study. Analyses were also restricted to area-based measures of body mass index and socio-economic status as this data is not currently available for individual patients.

The large variation in hospital rates of planned and intrapartum caesarean section found for Robson Group 5 in this study is consistent with previous work finding substantial inter-institutional variation in the mode of birth for deliveries among women with previous caesarean section and/or uterine surgery and a singleton pregnancy [42]. However, direct comparison of birth outcomes with this study is not possible due to differing definitions of a trial of labour and the lack of restriction to cephalic presenting term births.

Casemix heterogeneity within Robson Group 5 [10, 14, 15, 17] was hypothesised to substantially contribute to the between hospital variation. However, analysis according to the onset of labour and adjusting for case-mix factors including obstetric history, prior experience of labour, and prior vaginal birth and/or vaginal birth after caesarean only somewhat reduced the overall variation in planned repeat caesarean section rates (17.3 %). Although differences in casemix may be important in explaining variation in hospital caesarean rates, these findings suggest that hospital planned repeat caesarean section rates vary markedly for reasons other than individual’s characteristics. This finding is consistent with previous studies examining variation in caesarean section rates for other subgroups of the maternity population [11, 38].

Hospital-level factor adjustment of planned caesarean section rates, lead to a much greater reduction in variation (by a further 31.7 %). Similar to other studies [38, 43], the odds of both, planned caesarean section and intrapartum caesarean section, are increased for private hospitals compared to public hospitals, suggesting that there are other factors driving the decision for caesarean section at these hospitals, irrespective of whether labour is attempted. It is unknown whether these factors are associated with women’s or clinician’s management preferences. Other hospital factors associated with increased odds of planned caesarean section were an overall larger proportion of low risk deliveries at the hospital and a lower hospital propensity towards vaginal birth after caesarean. These factors suggest that there are features or cultural aspects of certain hospitals that influence the likelihood that a patient who has had at least one caesarean section will receive a planned caesarean section. Many guidelines recommend that a vaginal birth after a caesarean section only be offered in a hospital that has available resources for an immediate caesarean section [44–46], and so a high planned caesarean section rate may be appropriate if hospitals have difficulty accessing theatres or anaesthetic staff to perform rapid emergency caesarean section following a trial of labour after a caesarean section [47, 48]. Variation associated with hospital characteristics may be modifiable with hospital level interventions such as written guidelines for standardised management having been shown to be associated with an increase in the vaginal birth after caesarean section rate [49, 50]. Targeted hospital level interventions such as introduction of regular caesarean section audits and hospital funding tied to the hospital caesarean section rate have also been shown to increase vaginal birth after caesarean section rates with a concurrent downward trend in perinatal mortality and no change in maternal mortality [51]. There are currently no specific Australian guidelines for the management of multiparous women with at least one caesarean and a single cephalic pregnancy at term (Robson group 5). New South Wales state policy [52] recommends hospitals develop local guidelines, but the uptake, content and diversity of hospital-specific guidelines is unknown. Hospitals that used oxytocin among women with a previous caesarean section were more likely to achieve vaginal birth, consistent with previous work [53] and this factor may be modifiable, if appropriate.

Overall, only about half of the variation in hospital rates of planned caesarean section rates was explained by case-mix and hospital-level factors, suggesting that other important factors are not captured in these data. Patients’ and clinician’s preferences vary and may explain the remaining variation [54]. Attitudes and practices of physicians have been previously shown to strongly influence patient preferences in their choice of repeat caesarean section or VBAC [55], and may be modifiable, through interventions such as an educational strategy delivered by an opinion leader [56]. The remaining variability may also, in part, reflect uncertainty among physicians on how to best balance the benefits and risk of repeat caesarean section in women who have had a previous caesarean section [54]. In addition to being strongly influenced by practitioners, women’s preferences have been shown to be influenced by their individual experience and risk assessment, family commitments, safety concerns for the baby, their desire for predictability of birth and recovery, fear of labour or pain and a desire for sterilisation. [54]. An understanding of women’s preferences and physician’s decision-making is essential for the evaluation of quality and appropriateness of obstetric care provided to women [54] and warrants further investigation using a mixture of quantitative and qualitative approaches.

Rates of intrapartum caesarean section rates among women undergoing a trial of labour varied widely across hospitals. Following adjustment for case-mix, 16 of 73 hospitals had rates significantly different from the state average rate of intrapartum caesarean section following a trial of labour. This suggests that women with the same characteristics undergoing a trial of labour would have different risks of having an intrapartum caesarean section depending on the hospital attended. Not only is there variation between hospitals in being offered a trial of labour, the threshold to intervene once in labour appears to also differ. Hospital factors only explained a small proportion of the remaining variation. Notably, hospitals with high rates of instrumental delivery and VBAC for women after a caesarean for breech presentation had lower rates of intrapartum caesarean section. There is a trade-off with a higher instrumental delivery rate associated with lower odds of intrapartum caesarean section. These two factors may be modifiable and evidence for nulliparous women suggests that written guidelines for standardised labour management may provide an option to reduce the variation in intrapartum caesarean section rates between hospitals [50].

The appropriateness of caesarean section rates can be assessed by examination of the related morbidity and mortality. This study found higher rates of maternal and neonatal morbidity for a trial of labour compared to planned repeat caesarean section, consistent with previous work [57]. The observed higher rates of neonatal morbidity for an intrapartum caesarean section compared to a planned vaginal birth after caesarean are expected [57]. Similarly, the higher rates of postpartum haemorrhage at intrapartum compared to planned caesarean section are consistent with other studies [58]. Encouragingly, despite some increased variation for neonatal morbidity, lower rates of planned caesarean section were not associated with worse maternal or neonatal outcomes, suggesting that modification of rates should not have adverse effects. However, four outlier hospitals caused a triangular pattern of association between adjusted morbidity rates and quintiles of adjusted intrapartum caesarean section rates. Removal of these hospitals from analyses resulted in no significant associations, consistent with another study of women with previous caesarean section. That study found no differences between adjusted morbidity rates of obstetric residency program and non-residency program hospitals and rates of vaginal birth after caesarean section [48]. The reasons for the particularly high rates of morbidity for the outlier hospitals in the second and third intrapartum caesarean section quintiles are unknown and could be investigated via clinical audit. Hospitals identified in this study with low repeat caesarean section rates and low maternal and neonatal morbidities could also provide valuable insights for the improvement of maternity care.

## Conclusions

Hospital rates of planned and intrapartum caesarean section for women with at least one previous caesarean section vary widely and only some of the variation can be explained by case-mix and hospital-level factors, suggesting that additional factors influence practices. Hospital rates of planned caesarean section are not associated with morbidity, yet morbidity rates differed among quintiles of hospital intrapartum caesarean section rates in a non-systematic manner, influenced by a few outlying rates. Instituting hospital practice changes requires monitoring of morbidity to ensure no adverse effects.
